# Probing plasmonic nanostructures by photons and electrons

**DOI:** 10.1039/c4sc03508a

**Published:** 2015-02-11

**Authors:** Katrin Kneipp, Harald Kneipp, Janina Kneipp

**Affiliations:** a Danmarks Tekniske Universitet DTU , Department of Physics , 2800 Lyngby , Denmark . Email: kneipp@usa.net; b Humboldt Universität zu Berlin , Department of Chemistry , 12489 Berlin , Germany

## Abstract

We discuss recent developments for studying plasmonic metal nanostructures. Exploiting photons and electrons opens up new capabilities to probe the complete plasmon spectrum including bright and dark modes and related local optical fields at subnanometer spatial resolution. This comprehensive characterization of plasmonic properties of metal nanostructures provides new basic insight into the fundamental physics of “surface enhanced” spectroscopy in hottest hot spots and enables us to optimize plasmon supported processes and devices.

## Abstract 2

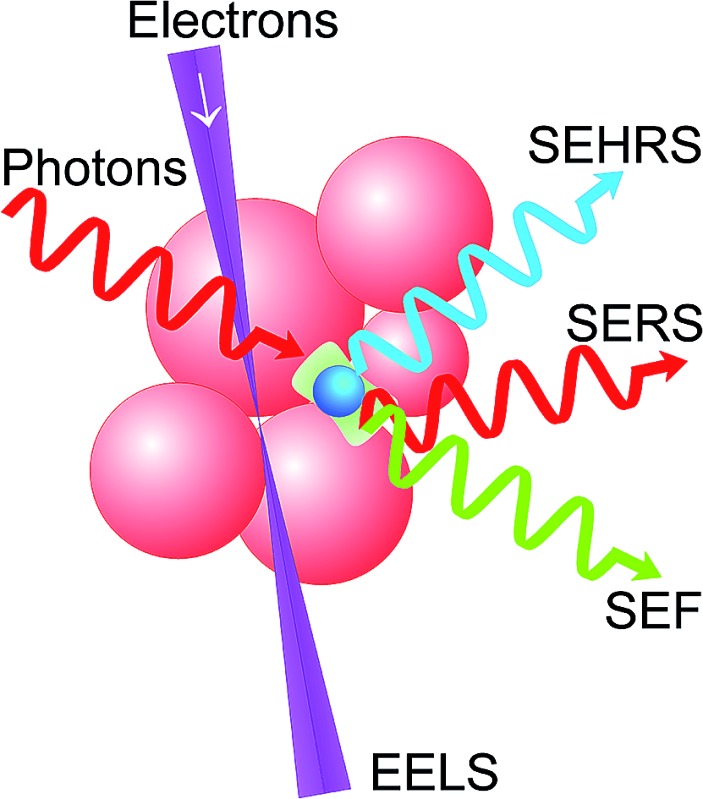
Exploiting photons *and* electrons opens up exciting new capabilities to study complex plasmonic nanostructures and related local fields.

## Introduction

Frequencies of collective oscillations of the free electrons in metal nanostructures, called surface plasmons, fall in the optical range of the electromagnetic spectrum.^1^ In complex metal nanostructures, plasmon hybridization results in a rich plasmon spectrum.^2,3^ Plasmon modes can be dipole active, leading to so-called bright modes, but plasmon oscillations can also give rise to dark modes which have a zero net dipole moment and usually do not show up in optical absorption experiments. Resonances between light and the surface plasmons result in enhanced optical fields in the vicinity of metal nanostructures. These high local fields have the potential to revolutionize optical spectroscopy and open up exciting new capabilities for almost all photon-driven processes.

Surface Enhanced Raman Scattering (SERS)^4^ is a prominent, example to demonstrate the power of plasmonic approaches. Raman spectra of a single molecule can be measured in the enhanced local fields of metal nanostructures.^5–8^ In particular, single molecule detection can be also achieved using non-resonant excitation in the near-infrared range.^5,8^ Giant SERS enhancement has been obtained only when composites of metal nanostructures such as aggregates of silver and gold nanoparticles were used as enhancing plasmonic structures.^5,7–10^ Interestingly, extinction spectra of plasmonic structures providing such a high enhancement level do not necessarily show resonances in the NIR, the range which was used for excitation when high enhancement is observed.^5,11^ This can be understood in the framework of the plasmonic properties of the enhancing aggregate structures. Theory predicts intense and extremely localized fields, for aggregates formed by metal nanoparticles, fractal structures and semicontinuous random metal films related to bright and dark plasmonic eigenmodes in these structures.^12–14^ Also in several other plasmonic structures, coupling between dark and bright modes, which results in the appearance of Fano resonances has been shown to be important for generating high local fields.^15–17^

Overall, the current experimental and theoretical insight shows that dark modes are of essential importance for the generation of highest local fields and for the spectroscopic performance of a plasmonic nanostructure. Therefore, comprehensive information on the plasmonic spectrum including bright and dark modes is of basic interest for deeper understanding of plasmon supported spectroscopy and for optimizing plasmon supported processes. Experimental tools for a comprehensive characterization of the plasmonic structures also have to address the strong confinement of their local fields. Specifically, SERS experiments indicate that highest enhancement level is available only for very few molecules^11^ residing in extremely small volumes. This means that the local confinement of hottest hot spots is in the sub-nanometer range.

In this minireview, we discuss methods for comprehensive probing of the plasmonic spectrum of metal nanostructures and of the related local fields. We start with considering optical experiments using one- and two-photon excitation. Tuned laser excitation enables to study the dependence of local fields on the photon energy. Spectroscopic signals collected from single molecules deliver sensitive information on the near field at the location of the molecule.

Complementary to photons, we discuss electron-energy-loss spectroscopy (EELS) as an emerging novel characterization tool for plasmonic nanostructures which can provide high spatial resolution at the subnanometer scale.^18–20^ In addition to excellent spatial resolution, EELS can probe bright and dark modes and, by this way, provides comprehensive information on the plasmonic spectrum.^21–26^

## Probing plasmon resonances and local fields using photons

Absorption and elastic scattering of light allow a straightforward probing of plasmon resonances. Beautifully colored glass windows in old cathedrals are related to scattering and absorption of sun light by surface plasmons of small metal nanoparticles incorporated into the glass matrix by old artists and masters hundreds of years ago. Today, hybridized surface plasmon resonances in aluminum nano disks and nanoholes can generate bright reflective colors to be used for coloration of plastic consumer products.^27^ Simple measurements in commercial spectrometers yield the extinction spectrum (losses due to absorption and scattering) of metal nanostructures. Usually, these measurements are performed on an ensemble of nanoparticles. For probing the plasmon spectrum of individual nanostructures absorption does not provide sufficient sensitivity, and collecting the scattered light using dark-field illumination is the more efficient experimental choice.

**Fig. 1:**
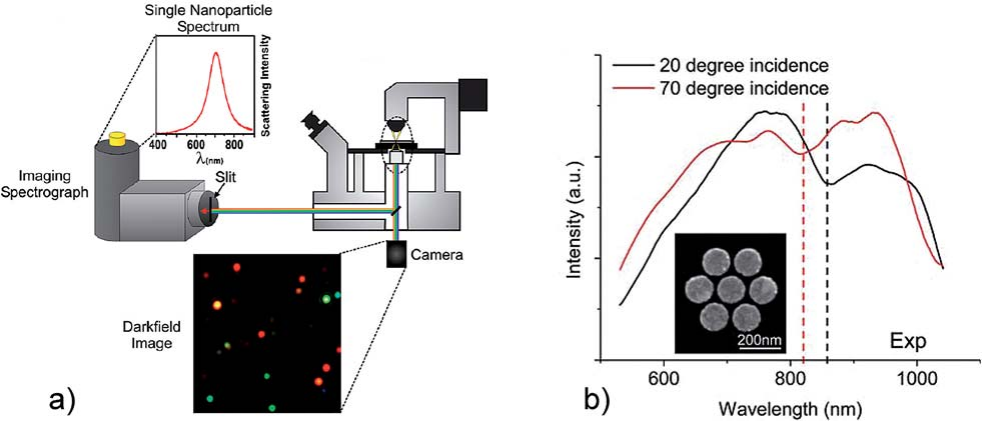
Probing the surface plasmon spectrum of individual nanostructures by dark-field microscopy. (a) Light scattering by different individual isolated silver nanoparticles. Adapted with permission from ref. 28, Copyright 2011 American Chemical Society. (b) Spectra of a plasmonic heptamer (see insert) measured for two different angles of illumination resulting in a shift of the Fano “dip”. Reprinted with permission from ref. 29, Copyright 2012 American Chemical Society.

Fig. 1a visualizes the plasmon resonances of individual silver nanoparticles of different size and shape which “glow” in different colors in a white light illuminated dark-field microscope. Combining this microscope with a spectrometer allows us to measure the plasmon spectrum of individual nanostructures.^28^

Usually, extinction probes bright plasmon modes. Dark modes become visible when they spectrally interfere with a bright mode and, by this way, borrow some oscillator strength from the bright mode. Such a Fano resonance is visible as an asymmetric dip in the spectrum around the energy of the dark mode. Sophisticated dark field microscopy which enables illumination with different incidence angle can identify shifts of Fano resonance minima in a nanoparticle aggregate in dependence on the angle of incidence of the excitation light.^29^ This provides insight into the bright and dark plasmon modes in the structure, and into their excitation mechanisms.

**Fig. 2:**
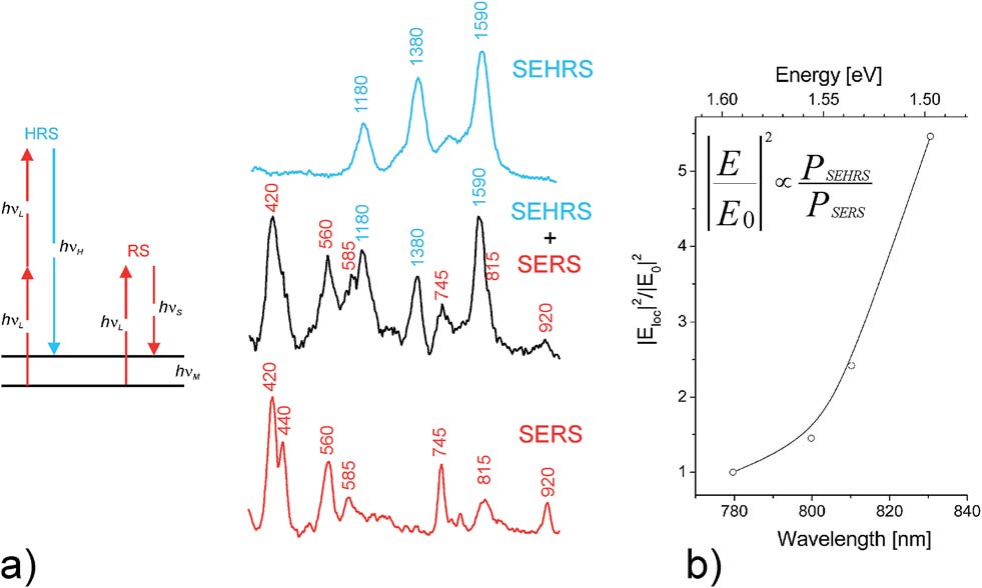
Probing local fields *E*_loc_ at the hottest spots of silver nanoaggregates using the ratio between SEHRS and SERS signals from single crystal violet molecules. (a) Schematic of one- and two-photon excited Raman scattering, RS and HRS, respectively as well as SEHRS and SERS spectra. Adapted with permission from ref. 9, Copyright 2006 American Chemical Society. (b) *E*_loc_^2^/*E*_0_^2^ in dependence of excitation wavelengths, where *E*_0_ is the optical field in the absence of the plasmonic structure. Adapted with permission from ref. 34, Copyright 2013 Springer.

In addition to knowing the complete plasmon spectrum including bright and dark modes, information on local optical field in the vicinity of a plasmonic structure is of basic scientific and practical interest. One- and two-photon excited spectroscopy performed in these local fields is a useful approach for getting this information. Spectroscopic signals collected from single molecules provide sub-nanometer probing of field amplitudes and polarization direction. For example, single molecule fluorescence has been exploited to map the local field of around a plasmonic structure.^30^ In case of one-photon excitation, the fluorescence signal quadratically depends on the local field amplitude. As a scattering process, SERS signals increase with the amplitudes of local optical field to the power of four.^31^ This makes SERS a very sensitive probe for local fields, particularly when also two-photon excited surface enhanced hyper Raman scattering (SEHRS) is applied. In SEHRS, two photons are scattered simultaneously resulting in a Raman signal shifted relatively to the second harmonic of the excitation laser^9,32^ (see Fig. 2a).

As a two-photon excited scattering process, SEHRS depends on the local field amplitudes to the power of six.^9,32^ Therefore, the ratio between SEHRS and SERS signals *P*_SEHRS_/*P*_SERS_ delivers direct information about local optical field intensities. Analyzing this ratio for different excitation wavelengths (Fig. 2b) reveals the dependence of local optical fields on the photon energy. The measured increase in intensity of local optical fields with increasing wavelengths as it is shown in Fig. 2b is qualitatively in agreement with computations of local fields for the hottest hot spots.^33^

Optical measurements as discussed in Fig. 2 deliver information on local optical fields from a (sub) nanometer probed volume, the location of a single probe molecule in the hot spot. However, due to a spatial resolution of about half of the applied wavelength, these experiments do not provide information where the hot spot is located on a plasmonic structure nor about the field distribution inside such a “hot area”.

**Fig. 3:**
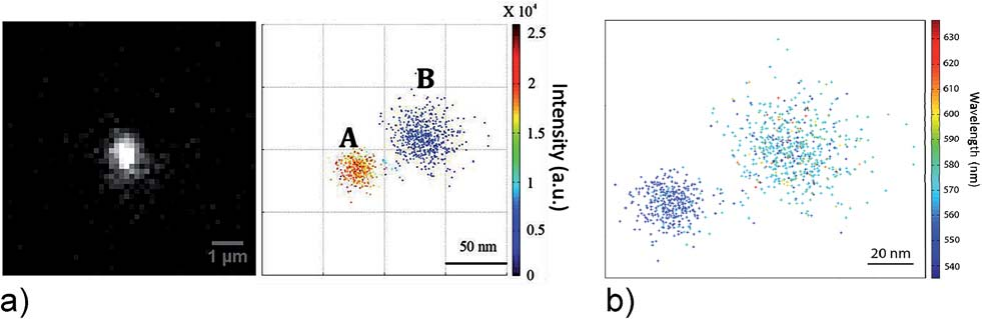
Probing local fields in hot spots of a silver nanowire–nanoparticle plasmonic structure using single-molecule fluorescence of Rhodamine B (a) fluorescence intensity image of two hot spots. (b) Image of the spectral distribution of individual dye molecules residing at different places in the hot spots. Reprinted with permission from ref. 36, Copyright 2013 American Chemical Society.

In order to get information on the location of hot areas around a plasmonic nanostructure and about distribution of local fields in these hot areas, sophisticated methods for super-resolution imaging and mapping beyond the diffraction limit have been developed based on surface-enhanced fluorescence^35,36^ and SERS signals.^37^ In these experiments, plasmon enhanced spectroscopic signals of single molecules are measured across the diffraction limited spot and localized by a two-dimensional Gaussian fit. By this way, emitters can be localized with a precision around 10 nm. Fig. 3 shows not only an intensity map of single fluorophores in hot areas, it also identifies spectral modulation of the fluorescence signal across the hot area.

The spatial distribution of surface enhanced Raman signals at the single molecule level can be used basically in the same way as fluorescence for the mapping of plasmonic local fields. Super resolution imaging of SERS hot spots including also the ability to obtain simultaneously spatial and spectral information is discussed in comprehensive recent reviews.^37,38^

Another method to measure the distribution of the local field intensity exploits changes in the decay rate of a fluorophore in the vicinity of a metallic nanostructure. This decay rate is proportional to the local density of plasmonic eigenstates which, in turn, is proportional to the intensity of the local field. Giant fluctuations in local fields of a fractal metal surface^39^ are identified by enhanced fluctuations in the decay rates of molecules.^40^

However, even sophisticated optical measurements cannot provide the spatial resolution for mapping out local optical fields with sub-nanometer resolution. As discussed in the next section, new approaches for probing plasmonic structures using fast electrons enable such experiments.

## Probing plasmon resonances and local fields using electrons

In general, the energy–momentum mismatch between freely propagating charges and photons prevents the linear coupling of fast electrons and light in vacuum. However, evanescent local optical fields carry the necessary complex momentum to make possible a coupling between freely propagating charges and photons. As an alternative to photons, surface plasmons can also be excited by low-energy^41^ and high-energy electrons.^42^ This opens up new ways for probing local fields of plasmonic nanostructures by electron energy loss spectroscopy (EELS).^19,20,43^

In an EELS experiment, a beam of monoenergetic fast electrons interacts with the plasmonic nanoparticles. The electric field associated with the fast flying electrons affects the free electrons in the metal nanostructure and generates a charge displacement, leading to a collective oscillation of conduction electrons at their plasmon resonance frequency. Due to this plasmon excitation, the fast electrons are losing the respective amount of energy. The energy spectrum of the electron beam after passing the plasmonic structure provides information on the plasmonic spectrum.

The capability of fast electrons to lose energy to the collective oscillation of the conduction electrons in a thick metallic foil has been discussed for the first time more than 50 years ago.^44,45^ In 1982, the excitation of localized surface plasmons was observed in EELS experiments on aluminum nanospheres.^42^ Surface plasmon resonances of isolated nanospheres and dimers (two-sphere-systems) were measured as low-energy peaks in EELS spectra along with the bulk plasmons of the metal. In particular, it was shown that EELS signals spread outside the nanostructure and that well-defined different plasmonic eigenmodes can be excited depending on the location of the electron beam relative to a nanoparticle dimer.^46^

Fast electrons came into the focus of interest as a highly localized excitation source of surface plasmons in 2007.^18,19,47^ EELS performed in a scanning transmission electron microscope (STEM) allows imaging of surface plasmons at sub-nanometer resolution. A general formalism describing the interaction of fast charged particles and plasmonic samples was described.^20^ Note that the monochromatic electron beam has energies of hundreds of keV, while electron energy loss appears typically between 1 and 3.5 eV, since plasmon modes typically appear in the optical range of the electromagnetic spectrum between 400 and 1000 nm. Challenges in EELS experiments on plasmonic nanostructures are discussed in a recent comprehensive review article.^43^

**Fig. 4:**
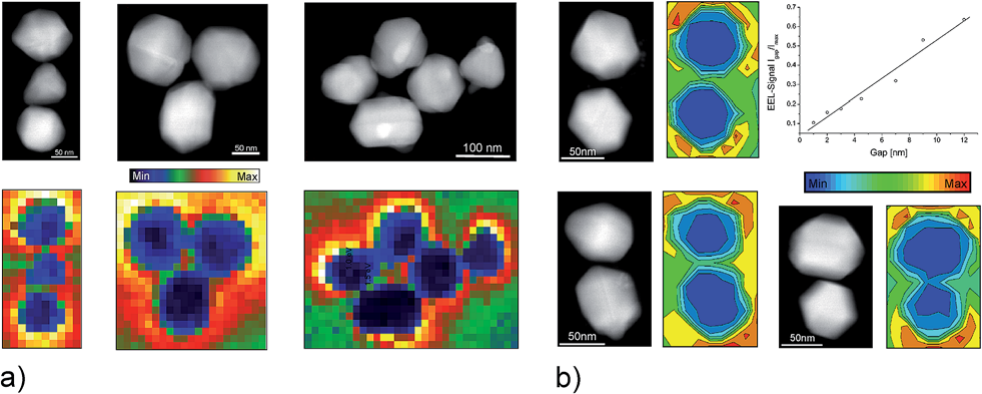
EELS probing of plasmonic nanoaggregates (a) EELS intensity maps of silver nanoaggregates generated in NIR energy windows of 1.6–1.8 eV (trimers) and 1.4–1.6 eV (five-particle aggregate). The plasmon resonance at 1.4–1.6 eV corresponds to the energy range used in Fig. 2. (b) EELS intensity maps for silver dimers with different gap sizes. The plot shows normalized EEL signals in the gap as a function of gap widths. Adapted with permission from ref. 34, Copyright 2013 Springer.

In general, the same optically active resonances, which determine the optical response of plasmonic structures, can be excited and studied by the electron beam in EELS. Since the electric field related to the fast electron varies at the scale of the nanostructure, *i.e.*, very rapidly compared to optical field, the electron beam also excites non-dipolar modes. The EELS spectra in Fig. 4 show this for plasmonic aggregates: in addition to a strong mode around 3.3 eV, which also dominates the absorption spectrum of silver nanoparticles, there exist modes in the NIR range. Excitation wavelengths used in one- and two-photon excited NIR-SERS experiments at extremely high enhancement level match these NIR plasmon resonances identified in the EELS studies.^34^ Also EELS spectra of discontinuous aluminum films show plasmon resonances in the NIR and strong SERS spectra have been obtained for these films at NIR excitation, even though the dielectric function of this metal supports the UV range.^48^

Images based on EELS intensities in the NIR range (see Fig. 4) show that areas of highest local fields correlate with those of lowest electron energy loss intensities. This is obvious by a comparison of EELS intensity maps and computations of local fields for dimers and trimers.^49,50,52^ The inverse relation between EELS maps and the local field distribution has been observed independently on specific geometries of nanoaggregates.^26,34,51^ Measurements on dimers with gap sizes between 12 nm and 1 nm (Fig. 4b) show a direct, almost linear relation between the EELS signal measured in the gap and the gap width. In contrast, computations for optical excitation show local field intensity enhancement proportional to 1/(gap size).^52^ The exact relationship between the electron energy loss and the local field strengths remains a subject of discussion.^53,54^

**Fig. 5:**
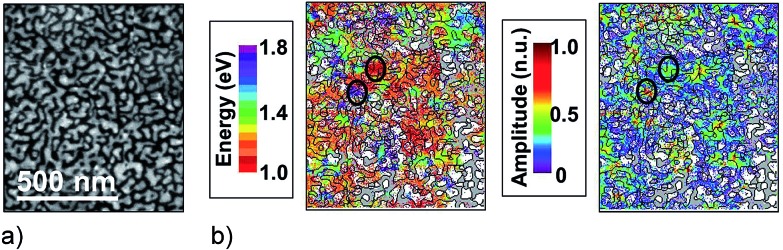
EELS probing of a silver semicontinuous film slightly before the percolation threshold (a) HAADF image. (b) Maps of the energy and amplitude of EELS peaks in the NIR between 1.0 and 1.8 eV (see color code, when no peak is found, the color is blanked if the local material is silver or greyed if there is silicon nitride). The superimposed contour line is obtained from the HAADF image. The black circles stress two different eigenmodes. Adapted with permission of ref. 57, Copyright 2013 American Physical Society.

Knowing the relationship between optically excited local fields and electron energy loss signals opens up the exciting possibility of probing local optical fields in the hottest hot spots and for experimental validation of computations that show dramatic spatial variations in local optical fields.^55,56^ For example, Fig. 5 provides some experimental evidence of the nanometer-scale confinement of plasmonic eigenmodes^39^ in random silver films around the percolation threshold which gives rise to extremely high hot spots on these structures.^57^

## Beyond classical and ultrafast

Usually, plasmonic nanostructures for creating high local fields are in dimensions between 10 and 100 nm, *i.e.*, in the case of metals, they are bulk materials that can be treated by classical theory. However, for very small particles below 5 nm or for small interparticle gaps in sub-nanometer dimension, plasmonic structures can exhibit also quantum nature.^25,58–64^ New experimental methods for probing plasmonic systems at high energy and spatial resolution enable us to observe such quantum effects. In individual metal nanoparticles, quantum effects result in characteristic blue shifts of the dipolar plasmon resonances.^58,59^ For nanoparticle dimers with narrow gaps, theory predicts the appearance of new charge transfer plasmon modes, as well as a breakdown of high local fields in the gap as hallmarks of quantum nature.^65^ EELS spectra collected from individual dimers with decreasing gap sizes down to atomic dimension allow to monitor the evolution of these modes.^25,60^ They are related to the onset of tunneling and, when the two spheres start to touch, to an oscillating current through a nanobridge between them. The classical bright and dark modes of the dimer should disappear with decreasing gap widths. Interestingly, silver dimers with atomic scale gaps can exhibit a regime, in which charge transfer modes and classical modes co-exist.^25^ The preservation of classical dipolar gap modes in the quantum regime when tunneling occurs can have important implications for local fields of plasmonic nanostructures in sub-nanometer gaps. In optical experiments, while SERS enhancement for a “classical” plasmonic dimer increases with decreasing gap widths,^10^ a decrease of the enhancement has been reported for gaps below 6.7 Angstrom when quantum effects emerge.^66^

Particularly exciting capabilities for probing plasmonic structures are opened up by experiments where fast electrons interact with surface plasmon fields which are pumped by incident photons. Optical pumping of plasmons gives rise also to an electron energy gain signals.^67^ Such experiments using loss and gain allow to combine the spatial resolution of electron microscopy with energy resolution as it can be achieved in optical excitation and probing. Moreover, today, not only light pulses but also electron pulses can be generated at femtosecond duration. This allows to probe photon excited plasmonic fields by electrons with temporal resolution at the time scale of the plasmonic fields.^68^ By exploiting all these capabilities, a new type of electron spectroscopy so-called photon-induced near-field electron microscopy (PINEM) has been invented.^69,70^ Experiments using PINEM enable space-time imaging of localized surface plasmon fields after excitation by incident photon pulses^71^ and provide basic insight into the nature of surface plasmons.

## Summary and outlook

Comprehensive information on plasmonic nanostructures and related local optical fields can be achieved by exploiting both photons and electrons. Their complementary use enables to access a new level of information by combining the high energy selectivity of laser radiation with the atomic scale spatial resolution of electron microscopy. Also, in the meantime, nanotechnology has allowed us to prepare tailored complex metal nanostructures such as nanoparticles at controlled size and shape or aggregates and composites with well characterized interparticle gaps down to atomic dimension.^10,66^ These developments in spectroscopy and nanotechnology provide us with tools and targets for comprehensive experimental studies of plasmonic excitations and related local fields. New capabilities to characterize plasmonic properties might become of particular interest for structures where quantum effects play a role, such as metal nanoaggregates with atomic scale interparticle gaps. For example, for dimers with subnanometer gaps, EELS and optical spectroscopy can indicate the onset of quantum effects.^25,60,66^ Composites of nanoparticles with very narrow gaps at the transition from the classical to the quantum regime seem to provide the highest local fields.

In the future, exploiting photons and electrons to study complex plasmonic structures will allow us to probe their complete plasmon resonance spectrum, to generate maps of local fields and hot spots at subnanometer resolution, and to determine local field intensities as a function of photon energy. These studies are of basic interest for the deeper understanding of the physics behind plasmonics and for enhancing and optimizing plasmon supported spectroscopy and other photon-driven processes.
